# Effectiveness and safety of transarterial chemoembolization combined with PD-1 inhibitors and lenvatinib for unresectable intrahepatic cholangiocarcinoma

**DOI:** 10.1186/s41747-025-00563-4

**Published:** 2025-02-18

**Authors:** Jin-Tao Huang, Di Hu, Xin Hong, Wen-Jie Zhou, Jian Shen, Peng-Hua Lv, Xiao-Li Zhu

**Affiliations:** 1 Department of Interventional Radiology, The First Affiliated Hospital of Soochow University, Suzhou, China; 2 Department of Interventional Radiology, Affiliated Hospital 2 of Nantong University, Nantong, China; 3 Department of Interventional Radiology, Northern Jiangsu People’s Hospital, Clinical Medical College of Yangzhou University, Yangzhou, China

**Keywords:** Camrelizumab, Chemoembolization (therapeutic), Cholangiocarcinoma, Lenvatinib, Tislelizumab

## Abstract

**Background:**

The objective of this study was to evaluate the therapeutic effectiveness and safety of transarterial chemoembolization (TACE) combined with programmed cell death-1 (PD-1) inhibitors and lenvatinib in the treatment of unresectable intrahepatic cholangiocarcinoma (uICC).

**Methods:**

This multicenter retrospective study screened patients with uICC who underwent TACE in combination with PD-1 inhibitors and lenvatinib between January 2019 and June 2023. Tislelizumab or camrelizumab (200 mg) was intravenously administered every three weeks. The daily dose of lenvatinib was 8 mg for patients weighing < 60 kg and 12 mg for those weighing ≥ 60 kg. In cases of disease progression, the therapeutic strategy was adjusted based on the clinical condition and individual patient’s treatment preferences. Options included transitioning to standard or supportive care or continuing treatment with TACE in combination with PD-1 inhibitors and lenvatinib. The primary outcomes were overall survival (OS) and progression-free survival (PFS), while secondary outcomes included the objective response rate (ORR), disease control rate (DCR), and the incidence of adverse events (AEs).

**Results:**

A total of 59 patients with uICC were included. Over a median follow-up period of 32.3 months, the median OS and PFS were 25.8 months (95% confidence interval [CI]: 17.9–33.7) and 9.5 months (95% CI: 7.9–11.0), respectively. The ORR was 55.9%, and the DCR was 96.6%. Grade 3 or four AEs were observed in 15 of 59 patients (25.4%).

**Conclusion:**

TACE combined with PD-1 inhibitors and lenvatinib demonstrated a promising therapeutic potential with a manageable safety profile for patients with uICC.

**Relevance statement:**

The combination of TACE, PD-1 inhibitors, and lenvatinib represents a novel therapeutic option for patients with uICC.

**Key Points:**

- TACE plus PD-1 inhibitors and lenvatinib represent a promising therapeutic strategy for uICC.
- The safety profile of TACE plus PD-1 inhibitors and lenvatinib was manageable.
- This study demonstrated improved outcomes compared to prior standard-of-care treatments.

**Graphical Abstract:**

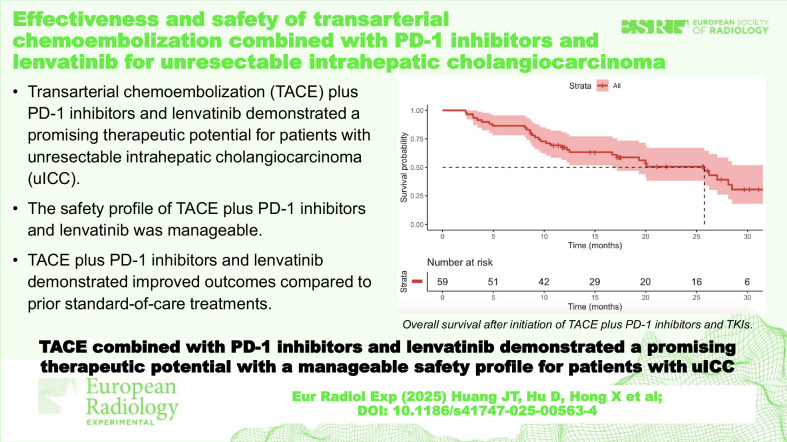

## Background

Intrahepatic cholangiocarcinoma (ICC) is the second most common malignancy among primary liver cancers, with a globally increasing incidence rate [1]. Surgical resection remains the only potentially curative treatment for resectable ICC [2, 3]. However, a substantial proportion of patients (54–70%) present with locally advanced or metastatic disease at the time of diagnosis, rendering them ineligible for surgical intervention [2, 4]. The phase III ABC-02 trial established the combination of gemcitabine and cisplatin as the standard treatment regimen for patients with advanced ICC [5].

Transarterial chemoembolization (TACE) is a treatment modality for unresectable ICC (uICC) that allows the localized delivery of high concentrations of chemotherapeutic agents to tumor sites, inducing ischemia. TACE is utilized as a locoregional tumor control strategy, either as adjuvant therapy following surgical resection or for patients with disease progression after initial treatments [6]. Recent advancements in genomic sequencing have facilitated the identification of genetic mutations, fueling interest in the development of targeted therapies and immunotherapies for ICC [7]. Tyrosine kinase inhibitors (TKIs) and programmed cell death-1 (PD-1) inhibitors have demonstrated efficacy in managing ICC, highlighting their potential in expanding treatment options for this malignancy [7–9].

Previous studies have reported the effectiveness of combining TACE with PD-1 inhibitors and TKIs, yielding favorable survival outcomes for hepatocellular carcinoma [10, 11]. Additionally, the combination of hepatic arterial infusion chemotherapy with PD-1 inhibitors and TKIs has shown efficacy and safety in uICC treatment [12]. However, the investigation of TACE combined with PD-1 inhibitors and TKIs for uICC remains limited [13]. Thus, this study sought to evaluate the therapeutic efficacy and safety of TACE combined with PD-1 inhibitors and lenvatinib for uICC in a real-world clinical setting, providing preliminary evidence of its potential benefits.

## Methods

### Patient criteria

The study was conducted in strict adherence to the ethical principles outlined in the Declaration of Helsinki and received approval from the institutional review board of The First Affiliated Hospital of Soochow University (date: 2024.07.10, no. 2024-361). Due to its retrospective design, the requirement for written informed consent was waived. Patients with uICC who underwent TACE combined with PD-1 inhibitors and lenvatinib between January 2019 and June 2023 were screened and included from three collaborating medical centers: the First Affiliated Hospital of Soochow University (Suzhou, China); Affiliated Hospital 2 of Nantong University (Nantong, China); and Northern Jiangsu People’s Hospital, Clinical Medical College of Yangzhou University (Yangzhou, China). The pathological diagnosis of ICC was established in accordance with current clinical guidelines [1], ensuring a standardized and rigorous approach to patient evaluation. Following the confirmation of an ICC diagnosis and the completion of all necessary diagnostic testing, patients and their families engaged in a decision-making process to determine the most appropriate therapeutic strategy. For the choice between standard treatment (gemcitabine and cisplatin) and the combination therapy of TACE with PD-1 inhibitors and lenvatinib, patients or their designated relatives, taking into consideration factors such as medical insurance coverage and complimentary drug policies, opted for the latter treatment regimen. The final decision regarding the treatment approach was made by the patients or their designated relatives, ensuring an informed and personalized selection.

Inclusion criteria were as follows: (1) a confirmed diagnosis of uICC; (2) hepatic function categorized as Child–Pugh class A or B; (3) allowance for tumor recurrence following curative resection; (4) at least one measurable intrahepatic lesion [14]; (5) uICC extending into liver parenchyma with mass formation; and (6) initiation of PD-1 inhibitors and lenvatinib within one month of TACE. Exclusion criteria included: (1) contraindications to TACE; (2) concurrent malignancies; and (3) incomplete or insufficient data for evaluation.

### TACE procedure

All patients underwent drug-eluting bead TACE according to standardized protocols to maximize tumor control and minimize complications [15, 16]. Using the Seldinger technique, a 4-F RH catheter (Cordis, Miami Lakes, USA) was inserted into the proper hepatic artery via the femoral artery. Indirect portal angiography was performed through the superior mesenteric artery to assess hepatopetal portal flow. Hepatic artery identification was followed by digital subtraction angiograms and three-dimensional arteriography using cone-beam computed tomography (CT) to guide catheter navigation.

After super-selective catheterization, cone-beam CT was employed to verify microcatheter positioning (2.4-F–2.7-F Renegade, Boston Scientific, Marlborough, USA). A mixture of CalliSpheres® (Hengrui Callisyn Biomedical Co., Ltd, Suzhou, China) microspheres and nonionic contrast medium was administered into tumor-feeding arteries at a rate of 1 mL/min. CalliSpheres microspheres (100–300 μm) were loaded with 50 mg of epirubicin as the chemoembolization agent. The TACE endpoint was defined as flow stasis in the tumor-feeding arteries, confirmed by fluoroscopy when contrast medium clearance was observed after 3–5 heartbeats. Repeated TACE was performed on a demand basis for patients with viable tumors identified through contrast-enhanced magnetic resonance imaging or CT during follow-up. All patients received 50 mg of epirubicin during the TACE procedure, with no residual vascular tissue observed.

### PD-1 inhibitors and lenvatinib administration

PD-1 inhibitors (tislelizumab/camrelizumab) and lenvatinib were administered within one month following TACE. Tislelizumab or camrelizumab (200 mg) was intravenously administered every three weeks, as recommended. The daily dose of lenvatinib was 8 mg for patients weighing less than 60 kg and 12 mg for those weighing 60 kg or more. In cases of disease progression, the therapeutic strategy was adjusted based on the clinical condition and treatment preferences of the individual patient. Options included transitioning to standard or supportive care or continuing treatment with TACE in combination with PD-1 inhibitors and lenvatinib.

### Assessment

Surveillance, conducted using contrast-enhanced CT and/or magnetic resonance imaging, was systematically scheduled at intervals of 2–3 months. Concurrently, laboratory evaluations were meticulously performed prior to each therapy and as part of regular follow-up visits. Regular follow-up continued until the death of the patient or the conclusion of the study on December 31, 2023. Imaging response evaluations were performed in accordance with the Modified Response Evaluation Criteria in Solid Tumors (mRECIST) [14] by two independent board-certified radiologists from each participating medical center. Safety monitoring was conducted throughout the follow-up period and was informed by laboratory test results and vital sign measurements. Adverse events (AEs) were classified using the Common Terminology Criteria for AEs (version 5.0) [17] in adherence to standardized guidelines.

### Outcomes

Primary outcomes included overall survival (OS) and progression-free survival (PFS). OS was defined as the time from therapy initiation to death from any cause. PFS was determined as the duration from the commencement of therapy to the first documented occurrence of disease progression, death, or the final follow-up assessment. Secondary outcomes included the objective response rate (ORR), disease control rate (DCR), and the incidence of AEs. ORR was calculated as the percentage of complete and partial responses, while DCR included complete responses, partial responses, and stable disease.

### Statistical analysis

Continuous variables were presented as medians with interquartile ranges, while categorical variables were expressed as frequencies and percentages. Statistical significance was determined using a two-tailed *p*-value threshold of < 0.05. All statistical analyses were performed using SPSS software, version 26.0 (IBM, Somers, NY).

## Results

### Patient characteristics

**Fig. 1:**
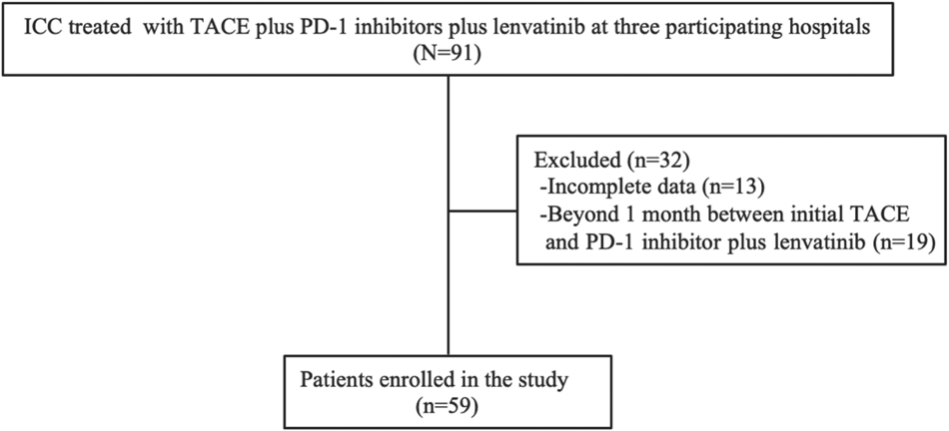
Flowchart of patient selection. ICC, Intrahepatic cholangiocarcinoma; PD-1, Programmed cell death-1 inhibitors; TACE, Transarterial chemoembolization

The study included 59 patients with histologically confirmed ICC, comprising 35 males and 24 females, as depicted in Fig. 1. Table 1 provides the baseline characteristics of the cohort. Of these patients, 45 (76.3%) had Child–Pugh class A status, while 14 (23.7%) had Child–Pugh class B. The median age was 66 years, ranging from 57 years to 71 years. Vascular invasion was observed in ten patients (16.9%), and extrahepatic metastasis was present in 21 patients (35.6%).

**Table 1:** Baseline characteristics of patients (*n* = 59)

Parameter data	Data
Epidemiology	
Age	66 (57–71)
Gender, males/females	35/24 (59.3/40.7)
Eastern Cooperative Oncology Group performance status	
0	43 (72.9
1	16 (27.1)
Child–Pugh stage	
A	45 (76.3)
B	14 (23.7)
Recurrence after surgery	
Yes	18 (30.5)
No	41 (69.5)
Tumor number	
Single	16 (27.1)
Multiple	43 (72.9)
Tumor size (cm)	
≤ 5	31 (52.5)
> 5	28 (47.5)
Vascular invasion	
Present	10 (16.9)
Absent	49 (83.1%)
Extrahepatic metastasis	
Present	21 (35.6%)
Absent	38 (64.4%)
Laboratory tests	
Total bilirubin [μmol/L]	16.7 (11.7–23.1)
Aspartate aminotransferase [U/L]	40.0 (22.0–60.0)
Alanine transaminase [U/L]	32.5 (18.0–53.0)
Platelet [10^9^/L]	164.0 (115.0–213.0)
Leukocyte [10^9^/L]	5.9 (4.6–7.1)
International normalized ratio	1.4 (1.3–1.6)
Neutrophil to lymphocyte ratio	3.3 (2.6–4.9)

All results are reported in the number of patients (%) or median with interquartile range unless otherwise stated

### Efficacy

**Fig. 2:**
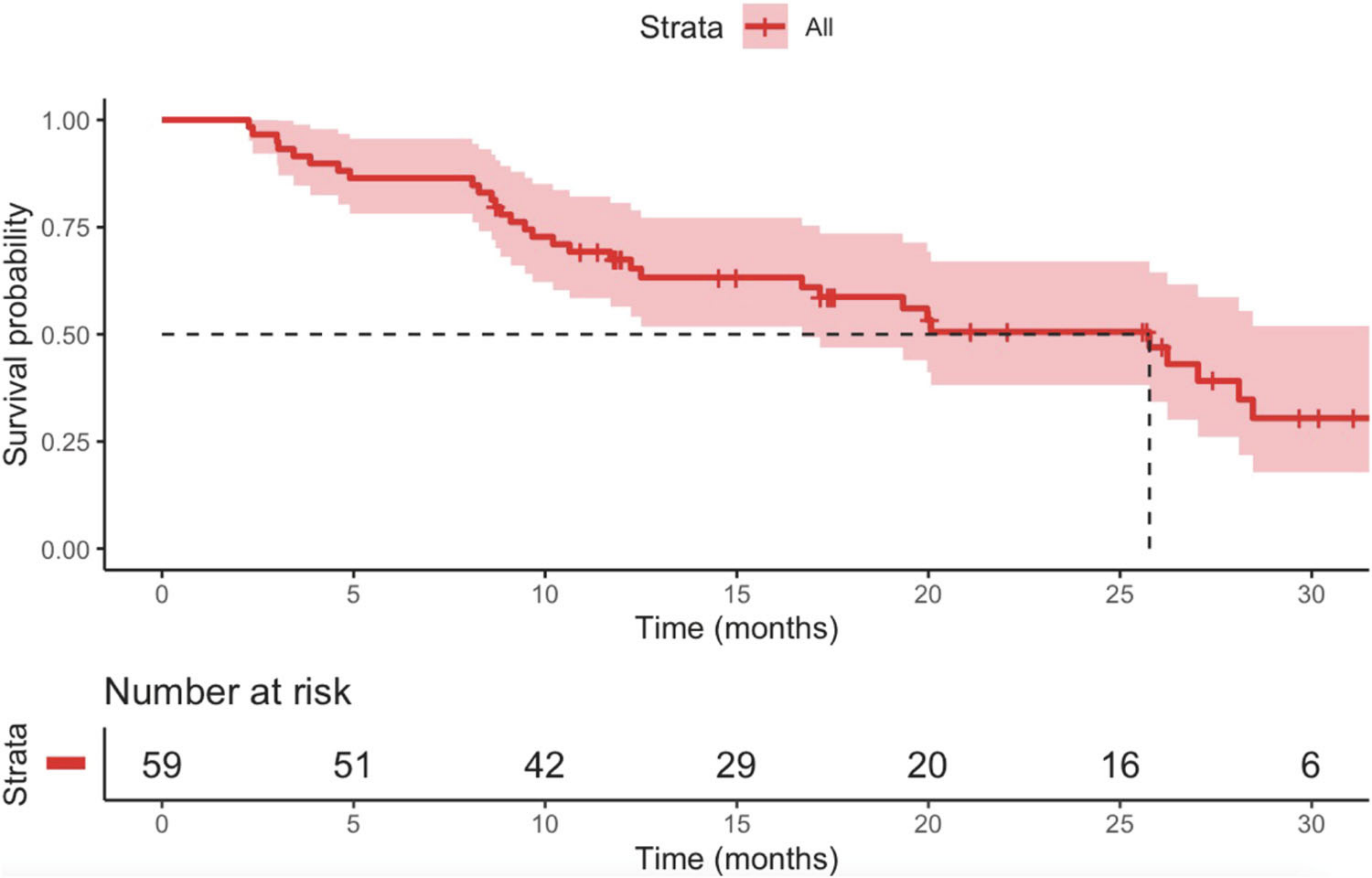
OS after initiation of TACE plus PD-1 inhibitors and lenvatinib

**Fig. 3:**
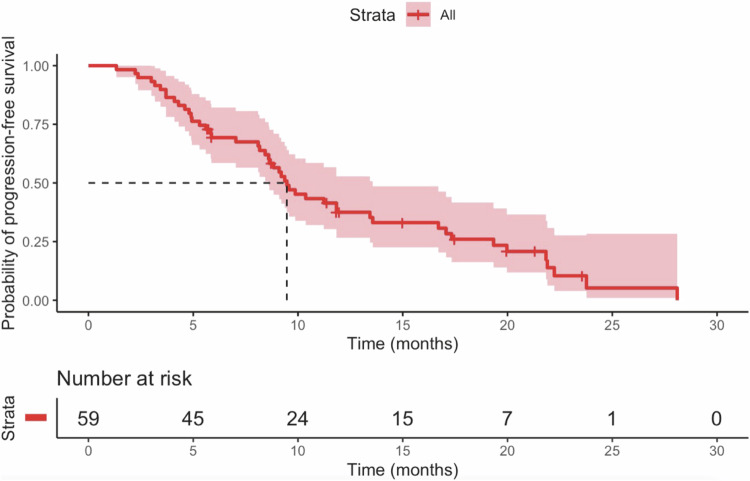
PFS curves after initiation of TACE plus PD-1 inhibitors and lenvatinib

**Fig. 4:**
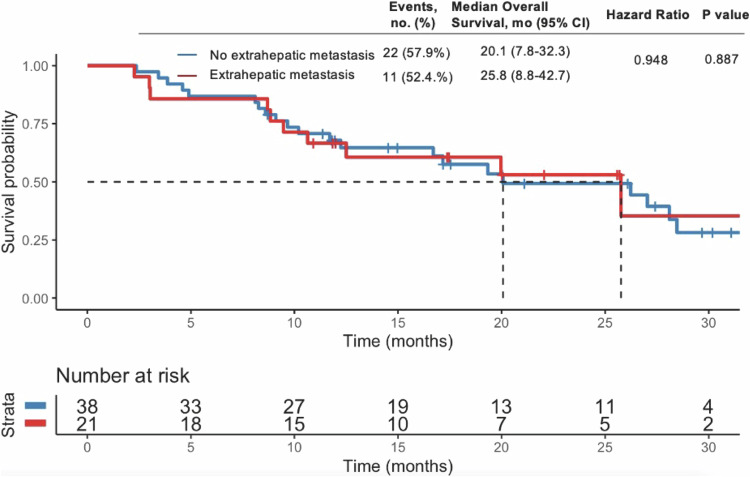
OS curves in patients with and without extrahepatic metastasis after initiation of TACE plus PD-1 inhibitors and lenvatinib

The median follow-up duration for the entire cohort was 32.3 months (95% confidence interval [CI]: 10.2–41.2). The median OS for the study population was 25.8 months (95% CI: 17.9–33.7) (Fig. 2), while the median PFS was 9.5 months (95% CI: 7.9–11.0) (Fig. 3). Patients without extrahepatic metastasis exhibited a median OS of 20.1 months compared to 25.8 months for those with extrahepatic metastasis (Fig. 4), although this difference was not statistically significant (*p* = 0.887). Table 2 summarizes the best response assessments. Three patients (5.1%) achieved a complete response, while 30 patients (50.8%) experienced a partial response. Stable disease was observed in 24 patients (40.7%), and progressive disease occurred in 2 patients (3.4%). The ORR and DCR were calculated to be 55.9% and 96.6%, respectively.

**Table 2:** Radiological response according to mRECIST

	Number (*n* = 59)	Percentage (%)
Best response		
Complete response	3	5.1%
Partial response	30	50.8%
Stable disease	24	40.7%
Progressive disease	2	3.4%
ORR	33	55.9%
DCR	57	96.6%

*mRECIST* Modified response evaluation criteria in solid tumors

### Safety

No instances of therapy-related mortality or unexpected serious AEs were reported. Table 3 provides a summary of grade 3 or 4 AEs in the 59 included patients following the initiation of TACE combined with PD-1 inhibitors and lenvatinib. The most commonly reported grade 3 or 4 AE was liver dysfunction (transaminitis) (*n* = 5, 8.5%), followed by nausea/vomiting (*n* = 3, 5.1%). Hypertension and hyperbilirubinemia were observed in two patients each, while fatigue, rash, and liver abscesses occurred in one patient each. Symptomatic treatments, including glucocorticoids, were administered based on the severity of the AEs and the organs involved. Following the implementation of symptomatic therapies, all AEs were resolved.

**Table 3:** Treatment-related AEs

Events	Grade 3 or 4 (*n*, %)
Fever	0
Nausea/vomiting	3 (5.1%)
Fatigue	1 (1.7%)
Cholangitis	0
Hematomas	0
Abdominal pain	0
Diarrhea	0
Rash	1 (1.7%)
Proteinuria	0
Hand-foot syndrome	0
Hypertension	2 (3.4%)
Liver abscess	1 (1.7%)
Hyperbilirubinemia	2 (3.4%)
Hypoalbuminemia	0
Liver dysfunction (transaminitis)	5 (8.5%)

## Discussion

This multicenter study reported a median OS of 25.8 months, a median PFS of 9.5 months, an ORR of 55.9%, and a DCR of 96.6%, along with manageable AEs. These findings indicate that the combination of TACE, PD-1 inhibitors, and lenvatinib is well-tolerated and achieves favorable outcomes for patients with uICC.

The median OS and PFS for advanced or metastatic cholangiocarcinoma treated with the standard therapy of gemcitabine plus cisplatin were 11.7 and 8.0 months, respectively [5]. The KEYNOTE-966 trial, which evaluated the combination of pembrolizumab with gemcitabine and cisplatin *versus* the standard gemcitabine-cisplatin regimen in advanced biliary tract cancer, reported a median OS and PFS of 12.7 months and 6.5 months, respectively [18]. Similarly, the TOPAZ-1 trial, which investigated durvalumab combined with gemcitabine and cisplatin in patients with untreated unresectable or metastatic biliary tract cancer, demonstrated a median OS of 12.8 months [19]. In contrast, previous studies of patients with ICC undergoing TACE reported median OS values ranging from 6 months to 30 months and median PFS values between 3 months and 17 months [4, 20–24]. Notably, Yang et al observed median OS and PFS of 13.3 months and 7.2 months, respectively, in uICC patients treated with TACE and PD-1 inhibitors [25]. The clinical outcomes in the current study surpass those reported in previous studies, suggesting that the combination of TACE, PD-1 inhibitors, and lenvatinib represents a promising therapeutic option for uICC. Furthermore, Ning et al reported on a similar triple-combination therapy involving TACE, PD-1 inhibitors, and lenvatinib. In their study of 14 patients with advanced ICC, they observed median OS and PFS of 27.3 months and 8.13 months, respectively, results that align closely with those of the present study [13]. Surprisingly, in this study, patients without extrahepatic metastasis exhibited a trend toward lower OS compared to those with extrahepatic metastasis, which may be attributed to the small sample size.

The ORR observed in this study was superior or comparable to those reported in the ABC-02 trial (26.1%), the KEYNOTE-966 trial (29%), and the TOPAZ-1 trial (26.7%). Similarly, the DCR exceeded those of the ABC-02 trial (81.4%), the KEYNOTE-966 trial (75%), and the TOPAZ-1 trial (85.3%). Moreover, the ORR and DCR were comparable to those reported in studies evaluating hepatic arterial infusion chemotherapy combined with PD-1 inhibitors and TKIs for uICC (ORR: 61.5%, DCR: 82.1%) [12]. These findings suggest that TACE, when combined with PD-1 inhibitors and lenvatinib, may exert synergistic antitumor effects and enhance local tumor control in uICC.

Advances in TACE technology, including the implementation of cone beam CT navigation, have significantly improved the accuracy of selective catheterization. Super-selective and meticulous TACE has enhanced the susceptibility of tumors to necrosis. However, TACE may induce tumor angiogenesis due to ischemia and hypoxia [26], highlighting the potential of combining TACE with TKIs to improve therapeutic efficacy. Pinato et al demonstrated that TACE reduced intratumoral densities of immune-exhausted effector cytotoxic and regulatory T cells, illustrating its pleiotropic effects on the tumor microenvironment and supporting the rationale for combining TACE with immunotherapy [27]. TACE-induced tumor cell necrosis releases tumor neoantigens, promoting the recruitment and activation of dendritic cells within the tumor microenvironment [28]. This transformation of an immunosuppressive microenvironment into an immune-supportive state may enhance the efficacy of systemic therapies. TKIs further contribute by transforming non-immunogenic “cold” tumors into inflamed “hot” tumors through microenvironmental remodeling [28]. Consequently, the combination of TACE with PD-1 inhibitors and lenvatinib likely generates synergistic antitumor effects, improving patient survival outcomes.

TACE also facilitates the targeted delivery of high concentrations of chemotherapeutic agents to tumors via arterial blood supply. Epirubicin, a commonly utilized agent, not only exerts cytotoxic effects on malignant cells but also induces strong immunogenic cell death, marked by the release of high mobility group box 1, calreticulin exposure, and ATP secretion, which potentiate antitumor immunity [29]. CalliSpheres microspheres enable high epirubicin loading, which may enhance their ability to activate antitumor immunity compared to microspheres loaded with other chemotherapeutic agents, further augmenting the efficacy of combination therapy.

Safety was a key aspect under consideration in this study. The predominant treatment-related AEs were liver function abnormalities, consistent with those observed in TACE-based therapies for hepatocellular carcinoma. No new or unexpected AEs were documented. All reported AEs were well-tolerated and aligned with the safety profiles reported in prior studies [5, 13, 25, 30]. Furthermore, all AEs were manageable with supportive treatments, and no treatment-related mortality occurred, underscoring the favorable safety profile of the combination therapy involving TACE, PD-1 inhibitors, and lenvatinib.

This study has several limitations that warrant consideration. First, the retrospective design introduces inherent biases, and the modest sample size limits the external validity and generalizability of the findings. Second, the absence of a control group receiving standard therapy represents another relevant limitation. To address this, comparative analyses with outcomes from prior studies utilizing standard treatment protocols were discussed. Finally, the use of two different PD-1 inhibitors in the study may have introduced variability in the results. This approach was adopted based on the similarity of their targets and was inspired by the concept of “umbrella” trials, which evaluate the efficacy of various drugs targeting specific genetic mutations within a single cancer type [31].

In conclusion, the combination of TACE, PD-1 inhibitors, and lenvatinib represents a promising and effective treatment strategy for uICC, demonstrating favorable efficacy and a manageable safety profile.

## Data Availability

The datasets used and/or analyzed during the current study are available from the corresponding author upon reasonable request.

## Abbreviations

AEs: Adverse events
CT: Computed tomography
DCR: Disease control rate
ICC: Intrahepatic cholangiocarcinoma
OS: Overall survival
ORR: Objective response rate
PD-1: Programmed cell death-1
PFS: Progression-free survival
TACE: Transarterial chemoembolization
TKIs: Tyrosine kinase inhibitors
uICC: Unresectable ICC
